# Coprevalence and associations of diabetes mellitus and hypertension among people living with HIV/AIDS in Cameroon

**DOI:** 10.1186/s12981-024-00624-5

**Published:** 2024-06-01

**Authors:** Peter Vanes Ebasone, Anastase Dzudie, Nasheeta Peer, Donald Hoover, Qiuhu Shi, Hae-Young Kim, Ellen Brazier, Rogers Ajeh, Marcel Yotebieng, Denis Nash, Kathryn Anastos, Andre Pascal Kengne

**Affiliations:** 1 Department of Medicine, University of Cape Town, Cape Town, South Africa; 2 Clinical Research Education Networking and Consultancy (CRENC), Yaounde, Cameroon; 3 Faculty of Medicine and Biomedical Sciences, University of Yaounde I, Yaounde, Cameroon; 4 Lown Scholars Program, Harvard T. H. Chan School of Public Health, Boston, USA; 5 Non-Communicable Disease Research Unit, South African Medical Research Council, Cape Town, South Africa; 6 Department of Statistics and Institute for Health, Health Care Policy and Aging Research, Rutgers the State University of New Jersey, New Brunswick, USA; 7 Department of Public Health, New York Medical College, New York, USA; 8 Institute for Implementation Science in Population Health, City University of New York, New York, NY USA; 9 Department of Epidemiology and Biostatistics, Graduate School of Public Health, City University of New York, New York, USA; 10 Ministry of Public Health, National AIDS Control Committee, Yaounde, Cameroon; 11 Division of General Internal Medicine, Department of Medicine, Albert Einstein College of Medicine/Montefiore Medical Center, Bronx, NY USA

**Keywords:** Hypertension, Diabetes, Cardiometabolic, Prevalence, HIV/AIDS, Cameroon

## Abstract

**Background:**

The association between HIV infection and increased cardiometabolic risk, attributed to chronic inflammation in people living with HIV (PLWH) and/or antiretroviral therapy (ART) effects, has been inconsistent. In this study, we aimed to assess the associations of HIV-related factors with hypertension (HTN) and type-2 diabetes mellitus (T2DM), and the potential mediation effects of body mass index (BMI) in the associations between ART use and HTN or T2DM in PLWH in Cameroon.

**Methods:**

A cross-sectional study was conducted with 14,119 adult PLWH from Cameroon enrolled in the International epidemiology Databases to Evaluate AIDS (IeDEA) between 2016 and 2021. HTN was defined as systolic/diastolic blood pressure ≥ 140/90 mmHg and/or current use of antihypertensive medication, while T2DM was defined as fasting blood sugar ≥ 126 mg/dL and/or use of antidiabetic medications. Univariable and multivariable multinomial logistic regression analyses examined the associations of factors with HTN alone, T2DM alone, and both (HTN + T2DM). Mediation analyses were conducted to assess the potential mediation roles of BMI, while controlling for age, sex, and smoking.

**Results:**

Of the 14,119 participants, 9177 (65%) were women, with a median age of 42 (25^th^–75th percentiles: 35–51) years. Age > 50 years was associated with HTN alone, T2DM alone, and HTN + T2DM compared to the age group 19–29 years. Men had higher odds of having HTN + T2DM. Overweight and obesity were predictors of HTN alone compared to being underweight. WHO stages II and III HIV disease were inversely associated with HTN alone compared to stage I. The odds of diabetes alone were lower with ART use. BMI partially mediated the association between ART use and hypertension, with a proportion of mediation effect of 49.6% (all p < 0.02). However, BMI did not mediate the relationship between ART use and diabetes.

**Conclusions:**

Traditional cardiovascular risk factors were strongly associated with hypertension among PLWH, while HIV-related exposures had smaller associations. BMI partially mediated the association between ART use and hypertension. This study emphasizes the importance of screening, monitoring, and managing HTN and T2DM in older, male, and overweight/obese PLWH. Further research on the associations of HIV disease stage and ART use with HTN and T2DM is warranted.

## Background

Antiretroviral therapy (ART) has significantly prolonged the life expectancy of people living with HIV (PLWH). With this increased longevity, PLWH are exposed to diseases of ageing, such as type 2 diabetes mellitus (T2DM) and hypertension (HTN), at risk levels similar to- or higher than those observed in the general population [1, 2]. Consequently, more PLWH are now dying from T2DM and HTN [3]. HTN and T2DM are closely interlinked and their coexistence in an individual markedly increases cardiovascular morbidity and mortality [4]. Additionally, HTN and T2DM commonly cluster with obesity, which is a major risk factor for both.

Increased cardiometabolic risks among PLWH are thought to result from increasing traditional cardiovascular risk factors and other effects of the HIV virus itself, and from ART [5–8]. HIV and ART potentially contribute to HTN and T2DM in PLWH through several biological processes including microbial translocation, chronic inflammation, adipogenesis and activation of the renin–angiotensin–aldosterone system, endothelial cell dysfunction, HIV related renal insufficiency and insulin resistance [9–12].

Among PLWH, evidence of the burden and association between HIV related factors and HTN and T2DM is equivocal and differs by region. Some studies observed a higher burden or association of HIV related factors (ART use, viral load and CD4 count) with HTN and/or T2DM [13, 14]; others observed a lower burden or no association [15]. The burden of coexistent HTN and T2DM and its association with HIV related factors in PLWH has not been extensively studied. Furthermore, in the analysis of causal pathways between ART use and HTN and/or T2DM, body mass index (BMI) for both has often been treated as a confounding covariate, whereas it may more appropriately be a mediator, [16]. It is important to clearly delineate the role of obesity on the association between ART use and HTN and T2DM among PLWH.

This is particularly pertinent in sub-Saharan Africa (SSA) where the burden of HTN, T2DM and other cardiovascular risk factors is rising steeply [17] in this region with the largest population of PLWH (> 25 million PLWH), three-quarters of whom use ART [18]. This includes Cameroon where, in 2022, there were 494,476 (2.7% prevalence) PLWH [19]. The burden of HTN and T2DM in Cameroon is equally high, with prevalence of HTN of 30.9% [20] and of T2DM of 5.8% [21] in the general population. We therefore sought to investigate the prevalence and co-prevalence of HTN and T2DM, their correlates and the mediation effect of body mass index (BMI) in the association of ART use with HTN and T2DM amongst PLWH in Cameroon.

## Methods

### Study design and setting

Data from Cameroon, collected for the International epidemiology Databases to Evaluate AIDS (IeDEA) study, forms the basis for this analysis. The IeDEA is a global research consortium collecting observational data in 7 regions around the world. The Cameroon data included in the current study were collected from January 2016 to December 2021 and the study design has been described in detail previously [22]. Briefly, Cameroon IeDEA is part of the Central Africa IeDEA with three contributing sites across three urban towns. It is a longitudinal cohort study that collects prospective and retrospective data. Secondary data from patient records are supplemented by primary data collection from patients after obtaining informed consent. The current analyses use baseline cross-sectional data collected when participants were enrolled into the study. Ethical approval for the study was obtained from the Comité National Pour La Recherche en Sante Humaine (CNERSH) in Cameroon.

### Study participants and data collection

Participants were eligible for this analysis if they were HIV positive, at least 19 years old and not pregnant at or during 6 months after enrollment into the study. Patients coming for routine clinic visits were approached by a trained data collector. If they agreed to participate, written informed consent was obtained, and the interview conducted. Data collected included socio-demographic factors, clinical characteristics, CD4 count, HIV RNA viral loads, weight, height, current antiretroviral therapy-regimen and other treatment history around the time of study enrollment.

### Outcomes and other variables

Our outcome variables were HTN and T2DM. HTN was defined as Systolic Blood Pressure (SBP) ≥ 140 mmHg and/or diastolic Blood Pressure (DBP) ≥ 90. mmHg and/or current use of antihypertensive medication according to the European Society of Cardiology (ESC)/European Society of HTN (ESH) guidelines [23]. HTN diagnosis was based on Blood Pressure (BP) measures done within ± 6 months of the enrollment date. BP was measured following the standard procedure for Office BP measurement, as described in detail previously [22]. T2DM was defined as fasting blood glucose (FBG) ≥ 126 mg/dL or reported use of antidiabetic medications. Impaired fasting glucose was defined as FBG readings of 100–125 mg/dL. FBS measurements were done after at least an 8 h overnight fast [24].

Selection of confounding variables was based on the nearest available measurements taken within a 6-month period, either prior to or following the date of participant enrollment. They were categorized as: age (19–29, 30–49, ≥ 50 years), sex (male vs female), marital status (single, married, live with a partner, separated or divorced and widowed), education level (none, primary, secondary (1st–5th years), high school (completed at least 6th or 7th years), employment status (employed, unemployed), smoking status (never, current, former) and alcohol consumption (never, monthly or less, 2–3 standard drinks per month, ≥ 1 standard drink or more per week). BMI in kg/m^2^, was classified according to the WHO guidelines as: underweight < 18.5, normal 18.5–24.9, overweight 25.0–29.9 or obese ≥ 30.0 kg/m^2^) [25]. Height was measured to the nearest 0.1cm using a stadiometer, while weight was measured to the nearest 0.1kg using a calibrated scale. Selected data for the above variables was based on the closest measurements ± 6 months of the enrollment date.

HIV related factors were defined as follows: HIV/AIDS disease stages (I, II, III and IV) were based on the WHO classification [26]. ART use was defined as having received ART prior to enrollment into the study. Estimated duration of ART was the time between first ART start date to the date of enrollment into the study. Time since diagnosis was the time between first date of recorded or self-reported HIV positive test and date of enrollment into the study.

### Statistical analysis

Data were analysed using R^®^ Version 4.2.3 (15-03-2023) statistical program, (R Core Team). Median and 25^th^–75th percentiles were calculated for continuous variables while frequency, percentages and 95% CIs were calculated for categorical variables. Chi-square tests and Fisher’s exact tests compared proportions, while Wilcoxon rank sum test compared continuous variables.

Multinomial logistic regressions analyses investigated the associations of HTN alone, T2DM alone and the combination of both hypertension and diabetes (HTN + T2DM), in univariable and multivariable analysis, always versus none of the conditions as the reference group. Variables found to be associated with the outcomes in univariable analysis (p < 0.10), based on the Likelihood Ratio test (LRT), were included in the final multivariable models. Those that were collinear were excluded (variance inflation factors > 10). A p-value < 0.05 was considered statistically significant.

**Fig. 1:**
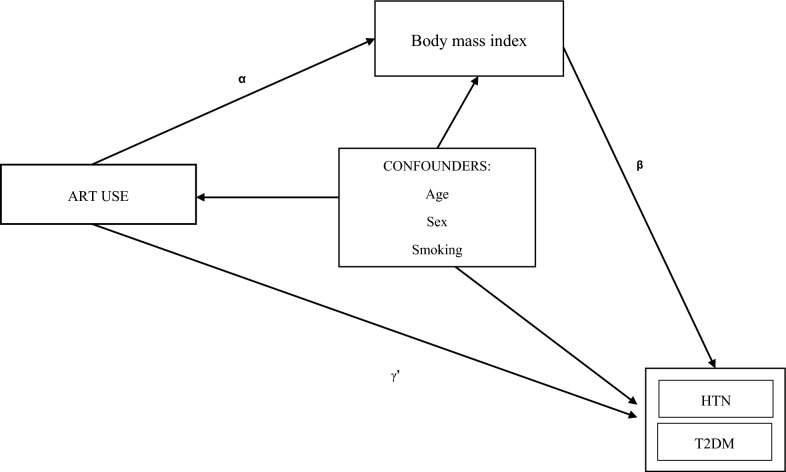
Schematic of BMI’s mediation of the relationship between ART use with hypertension or diabetes. Confounders are age sex and smoking. Path **αβ** represents the indirect effect while path **γ′** represents the direct effect. The total effect is the sum of paths **αβ + γ′.** *ART*   Antiretroviral therapy, *BMI*   Body Mass Index, HTN = hypertension, T2DM = Type 2 *diabetesmellitus*

Mediation analysis was performed to assess the mediation role of BMI in the association of ART use and T2DM or HTN. The rationale for the mediation analysis was based on the postulated pathogenetic mechanisms underlying the link between HIV and ART with cardiometabolic disease [9, 10, 12], and associations shown in previous studies [5, 6, 8, 27]. Figure 1 shows a schematic representation of the mediation role of BMI in the relationship between ART use with HTN or T2DM. The association between ART use [6, 8] and HTN or T2DM have been shown in previous studies (path γ**’**). Meanwhile, ART use [8, 28] has been reported to be associated with weight increases (path α). The positive association of adiposity with HTN and T2DM have been extensively described as well [29–32] (path β).

Mediation analysis was performed using the counterfactual-based mediation analysis framework by Imai et al. [33]. We used the Mediation package (version 4.5.0) in R [34]. The exposure was ART use (binary). The mediator was BMI (continuous) and the outcomes were either T2DM (binary) or HTN (binary) (Fig. 1). Two regression models were first fitted for each outcome, the mediator model using linear least squares regression and the outcome model using logistic regression, while controlling for confounders (age, sex and smoking) in both models. The average causal mediation effect (ACME), average direct effect (ADE), total effect and proportion mediated were then estimated through a nonparametric analysis bootstrapped in 1000 simulations. A sensitivity analysis was performed to examine the robustness of the mediation effect to the violation of the sequential ignorability assumption (absence of unmeasured confounders). The results of mediation analysis are reported according to the Guideline for Reporting Mediation Analyses Short-Form (AGReMA-SF) checklist [35].

## Results

### General characteristics of study participants

**Fig. 2:**
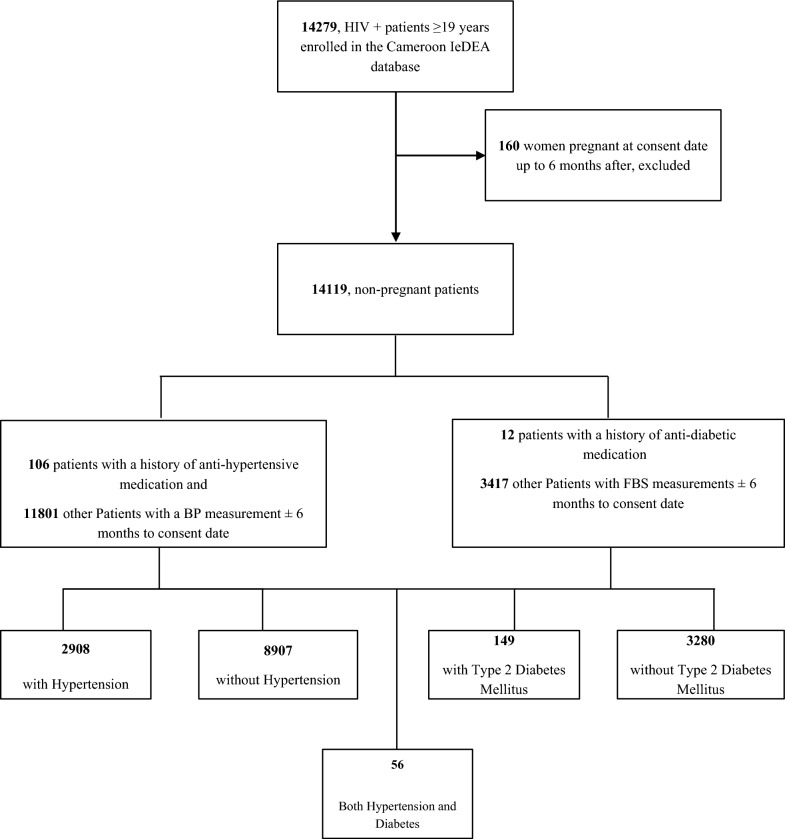
Study flow chart. IeDEA: International Epidemiology Databases to Evaluate AIDS

Of the 14,279 participants enrolled in the Cameroon IeDEA, 14,199 were eligible for this analysis (Fig. 2). As shown in Table 1, the median age (25^th^–75^th^ percentiles) of participants was 42 (35.0–51.0) years, and 9163 (65.0%) were women. Participants who had a viral load ≤ 200 copies/mL, and CD4 count ≥ 350 cells/mm^3^ were 4164 (78.9%) and 3289 (53.1%) respectively. Participants on ART numbered 8869 (63.1%), with 8869 (100%) using Nucleoside Reverse Transcriptase Inhibitors (NRTIs); 7402 (83.5%) using Non-Nucleoside Reverse Transcriptase Inhibitors (NNRTIs); 420 (4.7%) using Integrase Strand Transfer Inhibitors (INSTIs); and 823 (9.3%) using Protease Inhibitors (PI). Compared to those not on ART, participants on ART were mostly women, older, had longer times since HIV diagnosis and higher CD4 counts, and were more likely to be obese or overweight (all p < 0.001). However, those not on ART were more likely to be more educated, frequent drinkers and in WHO stages I and II (all p < 0.001) (Table 1).

**Table 1:** General characteristics of the study population by ART use status

Variable	Overall^§^, N = 14,058	ART use		p-value
		No, N = 5189	Yes, N = 8869	
Age in years, median (25^th^–75th percentile)	42 (35.0, 51)	38 (31.0, 46)	44 (38.0, 53)	
Missing	0	0	0	
Age category (in years), n (%)				< 0.001
19–29	1596 (11.4)	1055 (20.3)	541 (6.1)	
30–39	4019 (28.6)	1786 (34.4)	2233 (25.2)	
40–49	4559 (32.4)	1428 (27.5)	3131 (35.3)	
≥ 50	3884 (27.6)	920 (17.7)	2964 (33.4)	
Missing	0	0	0	
Sex, n (%)				< 0.001
Female	9131 (65.0)	3058 (58.9)	6073 (68.5)	
Male	4923 (35.0)	2130 (41.1)	2793 (31.5)	
Missing	4	1	3	
Highest level of education, n (%)				< 0.001
Never went to school	1407 (10.1)	533 (10.3)	874 (9.9)	
Primary	6129 (43.9)	2132 (41.3)	3997 (45.4)	
Secondary	3977 (28.5)	1509 (29.3)	2468 (28.0)	
High school or more	2455 (17.6)	982 (19.0)	1473 (16.7)	
Missing	90	33	57	
Smoking status, n (%)				< 0.001
Never smoked	11,706 (83.8)	4210 (81.7)	7496 (85.0)	
Current smoker	526 (3.8)	284 (5.5)	242 (2.7)	
Former smoker	1742 (12.5)	659 (12.8)	1083 (12.3)	
Missing	84	36	48	
Alcohol use, n (%)				< 0.001
Once a week or more	2081 (20.8)	1096 (24.3)	985 (18.0)	
2–3 times per month	1459 (14.6)	558 (12.4)	901 (16.4)	
Monthly or less	3315 (33.2)	1332 (29.6)	1983 (36.2)	
Never	3133 (31.4)	1,519 (33.7)	1614 (29.4)	
Missing	4070	684	3386	
Body mass index, n (%)				< 0.001
Underweight	890 (7.6)	503 (11.0)	387 (5.4)	
Normal weight	6092 (51.9)	2670 (58.1)	3422 (47.9)	
Overweight	3074 (26.2)	979 (21.3)	2095 (29.3)	
Obesity	1681 (14.3)	441 (9.6)	1240 (17.4)	
Missing	2321	596	1725	
HTN, % (95% CI)	24.6% (23.8–25.4)	19.2 (18.1–20.4)	28.0 (26.9–29.0)	< 0.001
Missing	2290	679	1611	
T2DM, % (95% CI)	4.3% (3.7–5.1)	5.6 (4.4–7.1)	3.7 (2.9–4.6)	0.009
Missing	10,637	4003	6634	
HTN + T2DM, % (95% CI)	1.8% (1.4–2.3)	1.4 (0.8–2.4)	2.0 (1.4–2.7)	0.282
Missing				
Impaired fasting glucose, % (95% CI)	17.8% (16.6–19.1)	15.2 (13.2–17.4)	19.3 (17.7–21.0)	< 0.001
Missing	10,637	4003	6634	
Time since HIV diagnosis in years,	3 (0.1, 7)	1.4 (0.8–2.4)	2.0 (1.4–2.7)	< 0.001
Missing	851	266	585	
WHO stage, n (%)				< 0.001
WHO Stage I	3243 (28.5)	1803 (36.9)	1440 (22.1)	
WHO Stage II	3113 (27.3)	1378 (28.2)	1735 (26.6)	
WHO Stage III	3952 (34.7)	1339 (27.4)	2613 (40.1)	
WHO Stage IV	1090 (9.6)	367 (7.5)	723 (11.1)	
Missing	2,660	302	2358	
ART duration in years, Median ((25th-75th percentile)	5 (1.4, 8)	- (-, -)	5 (1.4, 8)	
Missing	5,189	5,189	0	
CD4 count in cells/mm^3^, Median (25th-75th percentile)	373 (200.0, 565)	247 (100.0, 441)	414 (251.0, 596)	< 0.001
Missing	7,859	3,497	4362	
CD4 count level, n (%)				< 0.001
Above 350	3289 (53.1)	594 (35.1)	2695 (59.8)	
Less than 350	2910 (46.9)	1098 (64.9)	1812 (40.2)	
Missing	7859	3497	4362	
Log10 viral load in copies/mL, Median (25th-75th percentile)	2 (0.0, 2)	2 (0.0, 2)	2 (0.0, 2)	0.100
Missing	8781	4347	4434	
Viral load level in copies/mL, n (%)				0.957
> 200	1113 (21.1)	177 (21.0)	936 (21.1)	
≤ 200	4164 (78.9)	665 (79.0)	3499 (78.9)	
Missing	8781	4347	4434	

^***†***^^*1*^ n (%), ^***‡***^ Wilcoxon rank sum test; Pearson's Chi-squared test; Fisher's exact test. *ART* Antiretroviral therapy, *HTN* Hypertension, *T2DM*  Type 2 diabetesmellitus. ^**§**^61 participants were missing data on ART status

### Coprevalence of hypertension and diabetes

The prevalence (95%CI) of HTN, DM and HTN + T2DM were 24.6% (23.8–25.4), 4.3% (3.7–5.1) and 1.8% (1.4–2.3), respectively. The prevalence of HTN, T2DM and both HTN + T2DM by various sociodemographic and clinical factors is shown in Table 2. These prevalences were always higher in men versus women with 26.6% versus 23.5% (p < 0.001) for HTN, 6.1% versus 3.5% (p < 0.001) for T2DM and 2.4% versus 1.5% (p < 0.06) for HTN + T2DM. The prevalences of HTN, T2DM and HTN + T2DM significantly increased with older age with those 50 years and above having the highest prevalence. Former smokers, WHO stage I and obese participants also had the highest prevalence of HTN. The prevalence of HTN increased with higher BMI category. Patients with a CD4 above 350 cells/mm^3^ and a viral load < 200 copies/mL equally had a higher prevalence of HTN. Also, the prevalence of HTN was significantly higher among those who used ART versus ART-naïve (28.0% versus 19.2%), particularly INSTIs (33.3% versus 27.7%). There was generally a lower prevalence of diabetes with ART use (3.7% versus 5.6%), including NNRTI (27.8% versus 28.7%; p < 0.001) use (Table 2).

**Table 2:** Prevalence of hypertension, diabetes and combined hypertension and diabetes by sociodemographic and clinical factors

Characteristic	HTN (n = 2908) % (95% CI)	P-value	DM (n = 149) % (95% CI)	P-value	HTN + T2DM (n = 56) % (95% CI)	P-value
Age category		< 0.001		< 0.001		< 0.001
19–29	10.7 (9.1–12.5)		1.4 (0.4–3.7)		0.4 (0.0–2.5)	
30–39	16.3 (15.1–17.6)		3.0 (2.0–4.3)		0.9 (0.4–1.9)	
40–49	25.4 (24.1–26.8)		3.5 (2.5–4.8)		1.3 (0.8–2.3)	
≥ 50	38.3 (36.7–40.0)		7.5 (6.0–9.3)		3.5 (2.5–4.9)	
Sex		< 0.001		< 0.001		0.06
Female	23.5 (22.6–24.5)		3.5 (2.8–4.4)		1.5 (1.0–2.1)	
Male	26.6 (25.3–28.0)		6.1 (4.8–7.7)		2.4 (1.6–3.7)	
Education		0.003		0.03		0.05
Never went to school	28.8 (26.2–31.4)		6.2 (3.7–10.1)		2.1 (0.8–5.0)	
Primary	24.6 (23.4–25.8)		4.3 (3.4–5.5)		1.9 (1.3–2.8)	
Secondary	23.3 (21.9–24.8)		3.1 (2.2–4.3)		0.9 (0.5–1.8)	
High School or more	24.3 (22.5–26.2)		5.7 (4.1–8.0)		2.7 (1.6–4.5)	
Smoking		0.003		0.44		0.08
Never smoked	24.5 (23.6–25.3)		4.2 (3.5–5.0)		1.7 (1.2–2.3)	
Current smoker	19.3 (15.8–23.4)		4.5 (1.7–10.8)		0.0 (0.0–4.7)	
Former smoker	27.1 (24.8–29.4)		5.5 (3.5–8.3)		3.1 (1.6–5.7)	
Alcohol use		0.63		0.87		> 0.99
Never	22.9 (21.3–24.5)		4.6 (3.0–7.0)		1.9 (0.9–3.7)	
≤ 1 time/month	24.0 (22.5–25.6)		4.2 (3.1–5.8)		2.0 (1.2–3.2)	
2–3 times per month	24.0 (21.7–26.4)		5.0 (3.3–7.6)		1.9 (0.9–3.9)	
≥ 1 time/ week	24.4 (22.4–26.4)		5.0 (3.5–7.1)		2.0 (1.0–3.6)	
Body mass index		< 0.001		0.24		0.07
Underweight (< 18.5 kg/m^2^)	13.5 (11.3–16.1)		5.3 (2.8–9.5)		0.5 (0.0–3.3)	
Normal weight (18.5–24.9 kg/m^2^)	19.6 (18.6–20.6)		4.1 (3.2–5.3)		1.6 (1.0–2.5)	
Overweight (25.0 –29.9 kg/m^2^)	28.4 (26.8–30.1)		4.4 (3.2–6.2)		1.7 (1.0–3.0)	
Obesity (≥ 30.0 kg/m^2^)	41.7 (39.3–44.2)		6.3 (4.4–9.0)		3.3 (1.9–5.5)	
WHO stage		0.02		0.50		0.15
WHO Stage I	24.9 (23.3–26.6)		3.9 (2.7–5.6)		1.6 (0.9–2.9)	
WHO Stage II	22.1 (20.6–23.8)		4.8 (3.4–6.6)		2.6 (1.5–4.1)	
WHO Stage III	23.6 (22.3–25.1)		3.9 (2.9–5.1)		1.1 (0.7–2.0)	
WHO Stage IV	20.6 (18.1–23.3)		5.4 (3.5–8.3)		1.9 (0.8–4.1)	
ART use		< 0.001		0.009		0.28
No	19.2 (18.1–20.4)		5.6 (4.4–7.1)		1.4 (0.8–2.4)	
Yes	28.0 (26.9–29.0)		3.7 (2.9–4.6)		2.0 (1.4–2.7)	
NRTI						
Yes	28.0 (26.9–29.0)		3.7 (2.9–4.6)		2.0 (1.4–2.7)	
NNRTI		< 0.001		< 0.001		0.87
No	28.7 (26.2–31.4)		7.7 (4.9–11.8)		3.3 (1.5–7.0)	
Yes	27.8 (26.7–29.0)		3.2 (2.5–4.1)		1.8 (1.3–2.6)	
INSTI		< 0.001		0.09		> 0.99
No	27.7 (26.7–28.8)		3.6 (2.9–4.5)		1.8 (1.3–2.6)	
Yes	33.3 (28.1–39.1)		11.5 (3.0–31.2)		0.0 (0.0–22.9)	
PI		0.67		0.05		0.12
No	28.2 (27.2–29.3)		3.4 (2.6–4.3)		1.8 (1.2–2.6)	
Yes	25.3 (22.0–28.9)		7.2 (4.0–12.2)		3.6 (1.5–8.0)	
CD4 count		< 0.001		0.61		0.05
≥ 350 cells/mm^3^	27.6 (25.9–29.3)		3.9 (2.9–5.1)		2.4 (1.6–3.5)	
< 350 cells/mm^3^	21.9 (20.3–23.6)		4.3 (3.2–5.6)		1.3 (0.7–2.2)	
Viral load level in copies/mL		< 0.001		0.76		0.32
≥ 200	25.3 (22.6–28.1)		3.7 (1.6–7.8)		0.6 (0.0–3.7)	
< 200	30.9 (29.4–32.5)		4.2 (2.9–6.1)		2.0 (1.1–3.5)	

*HTN* Hypertension, *T2DM*   Type 2 diabetesmellitus, *ART*   Antiretroviral therapy, *INSTI*  Integrase strand transfer inhibitors, *NNRTI* Non-nucleoside reverse transcriptase inhibitors, *NRTI* Nucleoside reverse transcriptase inhibitors, *PI* Protease inhibitors. P-values represent Chi-square tests or fisher-exact tests where appropriate. P-values are for comparisons between HTN (Yes or No), T2DM (Yes or No) and HTN + T2DM (Yes or No) but only results for the Yes columns are shown

### Factors associated with hypertension and diabetes.

Table 3 presents the odds ratios (ORs) from univariable and multivariable multinomial logistic regression models (using participants known to have neither HTN nor T2DM as the reference group). In the adjusted model, the odds of HTN-alone (adjusted OR [aOR] 5.62; 95% CI [3.18, 9.95]), T2DM-alone (aOR 7.07; 95% CI [1.57, 32.0]), and HTN + T2DM (aOR 8.52; 95% CI [1.07, 67.8]) were significantly increased in participants over 50 years of age. Men had higher odds of HTN + T2DM compared to women (aOR 2.41; 95% CI [1.22, 4.77]). Overweight (aOR 2.07; 95% CI [1.10, 3.90]) and obesity (aOR 3.46; 95% CI [1.81, 6.64]) compared with underweight were also significantly associated with hypertension alone. In terms of HIV-related factors, participants with WHO stage II HIV disease (aOR 0.57; 95% CI [0.41, 0.79]) and WHO stage III (aOR 0.67; 95% CI [0.50, 0.90]) compared with WHO stage I were less likely to have hypertension alone in the adjusted analysis. Additionally, the odds of diabetes alone were decreased with ART use (aOR 0.44; 95% CI [0.22, 0.87]) Table 3.

**Table 3:** Logistic regression analysis for predictors of hypertension alone, diabetes alone, and both (N = 3133)

Variable	Unadjusted model				Adjusted model		
	n (%)	HTN-alone (n = 670)	T2DM-alone (n = 83)	HTN + T2DM (n = 56)	HTN-alone (n = 670)	T2DM-alone (n = 83)	HTN + T2DM (n = 56)
		OR (95% CI)^1^	OR (95% CI)^1^	OR (95% CI)^1^	OR (95% CI)^1^	OR (95% CI)^1^	OR (95% CI)^1^
Age (years)							
19–29	252 (8.0)	1.00	1.00	1.00	1.00	1.00	1.00
30–39	880 (28.1)	1.74 (1.08–2.81)*	3.27 (0.76–14.1)	2.49 (0.31–20.0)	1.75 (0.99, 3.10)	3.29 (0.75, 14.5)	0.97 (0.10, 9.47)
40–49	1055 (33.7)	2.95 (1.86–4.68)***	2.86 (0.66–12.3)	4.00 (0.52–30.6)	2.99 (1.70, 5.27)***	2.51 (0.54, 11.6)	2.93 (0.36, 23.9)
≥ 50	946 (30.2)	5.35 (3.38–8.47)***	7.90 (1.89–33.0)**	13.0 (1.77–95.8)*	5.62 (3.18, 9.95)***	7.07 (1.57, 32.0)*	8.52 (1.07, 67.8)*
Sex							
Female	2152 (68.7)	1.00	1.00	1.00	1.00	1.00	1.00
Male	980 (31.3)	1.04 (0.87–1.26)	1.66 (1.06–2.58)*	1.70 (1.00–2.91)	1.15 (0.91, 1.46)	1.65 (0.93, 2.94)	2.41 (1.22, 4.77)*
Education level							
Never went to school	242 (7.8)	1.00	1.00	1.00			
Primary	1352 (43.3)	0.87 (0.68–1.11)	0.76 (0.44–1.33)	0.97 (0.48–1.98)			
Secondary	969 (31.0)	1.11 (0.90–1.37)	1.56 (0.96–2.53)	1.85 (0.97–3.51)			
High school or more	559 (17.9)	1.02 (0.87–1.19)	1.01 (0.67—1.52)	1.75 (1.00–3.06)			
Smoking							
Never smoked	2,665 (85.5)	1.00	1.00	1.00			
Current smoker	99 (3.2)	1.31 (0.83–2.08)	2.14 (0.83–5.47)	0.65 (0.09–4.75)			
Former smoker	354 (11.4)	0.83 (0.62–1.11)	1.08 (0.55–2.13)	1.64 (0.82–3.29)			
Alcohol use							
Never	469 (20.4)	1.00	1.00	1.00			
≤ 1 time/month	857 (37.2)	0.89 (0.67–1.17)	0.72 (0.36–1.45)	1.00 (0.44–2.27)			
2–3 times per month	418 (18.2)	1.02 (0.74–1.40)	1.05 (0.48–2.26)	1.00 (0.38–2.63)			
≥ 1 time/ week	557 (24.2)	0.99 (0.74–1.34)	1.15 (0.57–2.32)	1.03 (0.42–2.52)			
Missing	832	1.01 (0.77–1.33)	0.72 (0.35–1.46)	0.68 (0.28–1.66)			
Body mass index (kg/m^2^)							
Underweight (< 18.5 kg/m^2^)	195 (7.1)	1.00	1.00	1.00	1.00	1.00	1.00
Normal weight (18.5–24.9 kg/m^2^)	1358 (49.3)	1.49 (0.94–2.36)	0.59 (0.28–1.24)	3.32 (0.44–24.8)	1.35 (0.73, 2.50)	0.52 (0.21, 1.29)	1.58 (0.20, 12.5)
Overweight (25.0–29.9 kg/m^2^)	748 (27.2)	2.25 (1.41–3.60)***	0.70 (0.31–1.56)	3.89 (0.51–30.0)	2.07 (1.10, 3.90)*	0.89 (0.33, 2.45)	1.26 (0.14, 10.9)
Obesity (≥ 30.0 kg/m^2^)	454 (16.5)	4.41 (2.73–7.11)***	0.89 (0.37–2.14)	9.28 (1.21–70.9)*	3.46 (1.81, 6.64)***	1.30 (0.43, 3.88)	3.97 (0.47, 33.4)
Missing	378	2.05 (1.24–3.38)**	0.32 (0.10–0.96)*	2.84 (0.33–24.5)	1.84 (0.96, 3.53)	0.26 (0.06, 1.04)	1.07 (0.11, 10.1)
WHO stage							
WHO Stage I	739 (24.7)	1.00	1.00	1.00	1.00	1.00	1.00
WHO Stage II	666 (22.3)	0.70 (0.54–0.90)**	0.98 (0.51–1.89)	1.46 (0.69–3.10)	0.57 (0.41, 0.79)***	1.17 (0.49, 2.79)	0.98 (0.37, 2.57)
WHO Stage III	1,224 (40.9)	0.76 (0.61–0.94)*	0.95 (0.53–1.69)	0.66 (0.30–1.43)	0.67 (0.50, 0.90)**	1.39 (0.62, 3.14)	0.57 (0.22, 1.52)
WHO Stage IV	362 (12.1)	0.77 (0.57–1.05)	1.23 (0.59–2.56)	1.13 (0.44–2.91)	0.68 (0.45, 1.02)	1.69 (0.62, 4.57)	1.25 (0.41, 3.82)
Missing	142	1.30 (0.87–1.94)	0.60 (0.14–2.62)	2.85 (1.04–7.78)*	1.23 (0.68, 2.25)	1.15 (0.14, 9.55)	2.23 (0.43, 11.4)
Time since HIV diagnosis (years)	3077	1.00 (0.96, 1.03)	0.92 (0.83, 1.02)	1.02 (0.92, 1.12)	1.00 (0.96, 1.03)	0.92 (0.83, 1.02)	1.02 (0.92, 1.12)
Duration of ART use (years)	2081	1.04 (1.01–1.07)**	1.02 (0.94–1.12)	1.06 (0.98–1.15)			
ART use							
No	1049 (33.5)	1.00	1.00	1.00	1.00	1.00	1.00
Yes	2081 (66.5)	1.38 (1.14–1.66)***	0.41 (0.26–0.63)***	1.45 (0.80–2.63)	0.92 (0.69, 1.22)	0.44 (0.22, 0.87)*	0.88 (0.37, 2.11)
NRTI							
No	1058 (33.8)	1.00	1.00	1.00			
Yes	2075 (66.2)	1.36 (1.13–1.65)**	0.41 (0.26–0.63)***	1.33 (0.74–2.39)			
NNRTI							
No	1263 (40.3)	1.00	1.00	1.00			
Yes	1870 (59.7)	1.35 (0.93–1.96)	0.35 (0.16–0.75)**	0.56 (0.24–1.28)			
PI							
No	2965 (94.6)	1.00	1.00	1.00			
Yes	168 (5.4)	0.58 (0.37–0.90)*	2.58 (1.11–6.01)*	0.58 (0.37—0.90)*			
CD4 count							
≥ 350 cells/mm^3^	1108 (50.2)	1.00	1.00	1.00	1.00	1.00	1.00
< 350 cells/mm^3^	1100 (49.8)	1.37 (1.11–1.69)**	0.60 (0.34–1.06)	1.99 (1.03–3.84)*	1.06 (0.83, 1.35)	0.80 (0.42, 1.54)	1.43 (0.68, 3.02)
Log10 viral load, copies/mL	817	0.86 (0.77–0.95)**	1.17 (0.93–1.47)	0.84 (0.59–1.20)			
Viral load level, copies/mL							
> 200	173 (21.2)	1.00	1.00	1.00			
≤ 200	644 (78.8)	1.61 (1.06–2.43)*	0.79 (0.28–2.26)	3.94 (0.51–30.3)			

^***1***^ *p < 0.05; **p < 0.01; ***p < 0.001. *OR* Odds Ratio, *CI* Confidence Interval, *LRT* Likelihood ratio test. *HTN* Hypertension, *T2DM* Type 2 diabetes mellitus, *ART* *Antiretroviral therapy*, *INSTI* Integrase strand transfer inhibitors, *NNRTI* Non-nucleoside reverse transcriptase inhibitors, *NRTI* Nucleoside reverse transcriptase inhibitors, *PI* Protease inhibitors. The reference group was made up of participants who were known to have neither hypertension nor diabetes. Only participants whose hypertension and diabetes status were known were included for this analysis, to ensure that the outcome categories are mutually exclusive. The participants were distributed as follows (HTN-alone = 670,T2DM-alone = 83, HTN + T2DM = 56, None (have neither HTN nor T2DM) = 2324 and Missing = 10986)

### Mediation and sensitivity analysis

Results of the mediation effect of BMI on the association of ART use and HTN or T2DM are shown in Table 4. After adjustment for age, sex and smoking, BMI partially mediated the association between ART use and HTN, with a mediation effect proportion of 49.6% (all p < 0.02). However, BMI had no mediating effect on the association between ART use and T2DM.

**Table 4:** Results of causal mediation analysis of the mediation effect of BMI on the association between ART use and HTN or DM, adjusted for age, sex and smoking

Outcome/Effect type	HTN			T2DM		
	Estimate	95% CI	p-value	Estimate	95% CI	p-value
Average mediation effect (indirect effect)	0.019	0.012 to 0.023	< 0.001	0.001	- 0.0003 to 0.0033	0.12
Average direct effect	0.020	0.003 to 0.035	0.014	- 0.037	- 0.058 to–0.018	< 0.001
Total effect	0.039	0.022 to 0.055	< 0.001	- 0.034	- 0.053 to–0.017	< 0.001
Percent of total effect mediated	49.6%	34.2% to 86.3%	< 0.001	- 4.0%	**- 13.3% to 1.1%**	**0.12**

*HTN*   Hypertension, *T2DM*   Type 2 diabetes mellitus, *ART*  Antiretroviral therapy

**Fig. 3:**
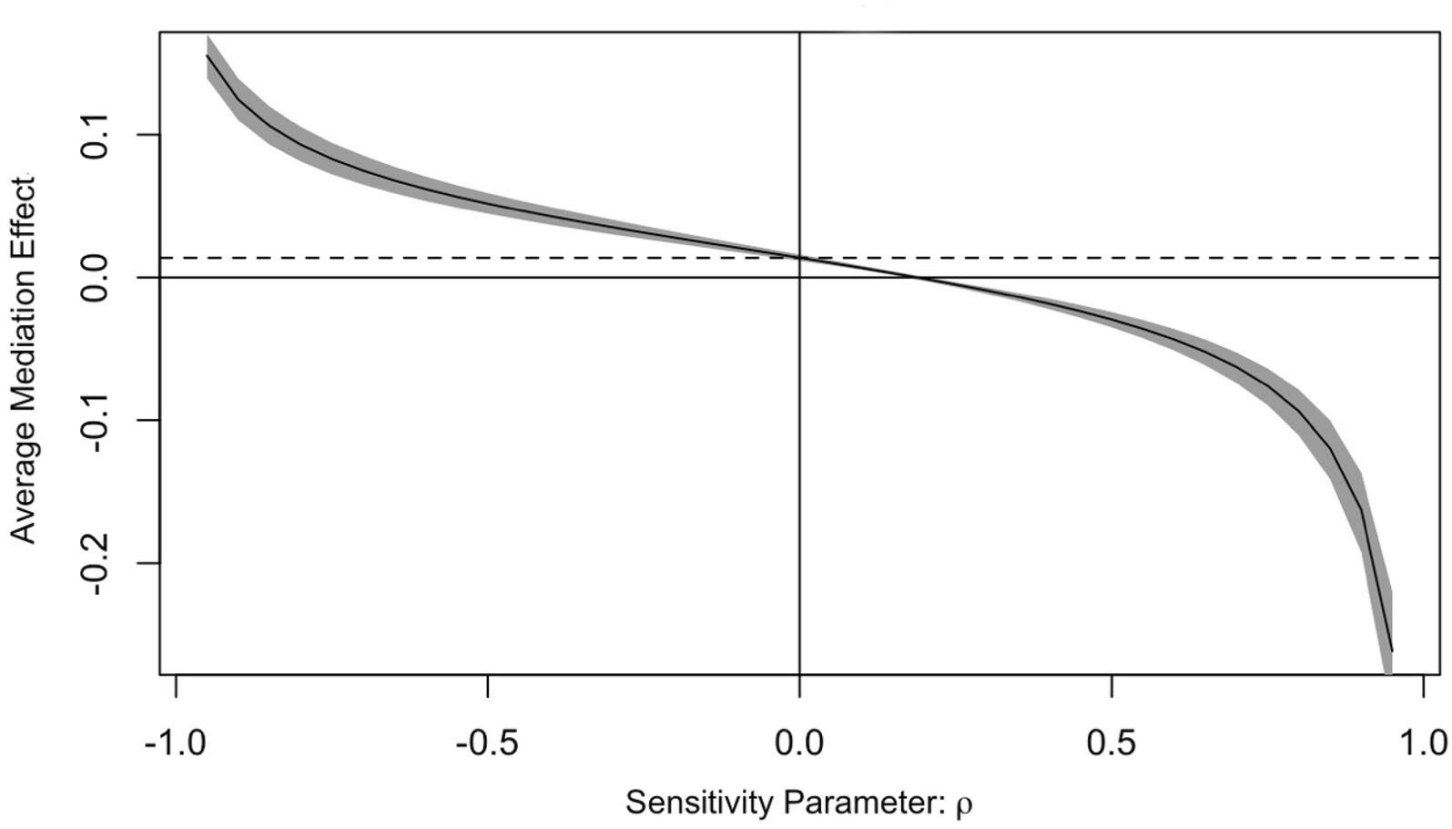
Sensitivity analysis of BMI’s mediation effect in the association between ART use and hypertension

To test the robustness of the causal mediation analysis (under the sequential ignorability assumption), a sensitivity analysis was done while adjusting for age, sex, and smoking (Fig. 3). In the analysis of the mediation effect of BMI on the association between ART use and HTN, it takes ρ = 0.2 to reduce the mediation effect to zero. The results of sensitivity analyses therefore suggest that the findings of the mediation analysis are quite sensitive to the violation of the sequential ignorability assumption. This means it would take a smaller unmeasured confounder(s) to overturn the conclusions obtained from the mediation analysis results.

In Fig. 3, the dashed line represents the estimated average mediation effect, while the solid line represents the estimated average mediation effect at different levels of rho (ρ), and the gray region represents the 95% confidence interval for estimated average mediation effect at different levels of ρ. The sensitivity parameter ρ denotes the correlation coefficient between the residuals of the mediator and outcome regressions models. It signifies the degree of unmeasured confounding in both regression models of the mediation analysis. Under the sequential ignorability assumption (ρ = 0), deviations from zero indicate how much effect of unmeasured confounding is needed to overturn the results obtained in the mediation analysis. If a small deviation in ρ from zero leads to a complete wipe out of the mediation effect, it indicates that the results are sensitive to the presence of unmeasured confounding (Fig. 3).

## Discussion

In this study involving 14,199 PLWH in Cameroon, the prevalence of HTN was substantial while the burden of T2DM was low to moderate, similar to the profiles in the general population. Older age, male sex, and higher BMI were associated with HTN and T2DM. However, we also found that WHO stages II and III HIV disease and ART use were associated with a lower prevalence of HTN and T2DM, respectively. Additionally, our analysis revealed that BMI had a partial mediation effect on the association of ART use and viral load with HTN, but not T2DM.

The prevalence of HTN in PLWH of 24.6% in the present study accords with that in PLWH in Cameroon [36, 37], and Uganda [38], and is comparable to the prevalence of 25.2% and 23.6% reported in two global systematic reviews in PLWH by Xu et al. [39] and Bigna et al. [14] respectively. It is however lower than the 30.9% prevalence found in a systematic review in the general population in Cameroon [20]. Our observed prevalence of T2DM in Cameroonian PLWH of 4.3% is comparable to the 3.8% reported by Rhee et al. [40] in PLWH in Cameroon, but lower than the 5.1% reported in a systematic review of PLWH in Africa [41] and 5.8% found in a pooled sample of 37,147 participants in the general Cameroon population [21]. The co-prevalence of HTN + T2DM of 1.8% was lower than the 3.3% observed in PLWH in Ethiopia [32] and the 6.9% in the general population in Cameroon [29]. The variability in the prevalence of HTN and T2DM could be attributed to differences in study population, study setting, screening and diagnostic criteria, the clinical stage of HIV/AIDS disease, and ART status or type.

The association of older age, male sex and overweight/obesity with HTN and T2DM in this HIV population is in keeping with what was reported in the general population in Cameroon [20, 21, 29]. Similarly, our findings agree with other studies [30–32, 42, 43] conducted in PLWH in SSA. This suggests that these traditional cardiovascular risk factors of older age, male sex and overweight/obesity are also the main drivers of HTN and T2DM in PLWH.

Participants with WHO stages II and III HIV disease, compared to those with stage I had 43% and 33% reduced odds, respectively, of HTN-alone. A study of 34,111 Highly Active Antiretroviral Therapy (HAART) naïve HIV-Infected adults in Tanzania reported a 12% and 28% reduced odds of HTN in participants with WHO stages II and III HIV disease respectively [30]. Since our study population included a substantial number of patients with advanced HIV disease, the inverse relation of HTN and immune suppression in this study is likely due to the association of lower BP with later stage HIV disease [44, 45].

The evidence for the relation between ART and diabetes is equivocal. While one systematic review described 4 times higher odds of diabetes with ART use [8], other reviews did not establish any significant association between the two [41, 46]. Conversely, the current study found an unexpected 56% reduced risk of diabetes alone with ART use which accords with another study in the country: Rhee et al. found 54% reduced odds of diabetes with ART use [40]. The mechanism behind the protective effect of ART on diabetes is unclear. It could be due to host response to infection or some other factor that could contribute to not being on ART, hence predisposing to diabetes [47]. Further research is warranted to clarify the association and the pathophysiological pathways.

In the current study, BMI partially mediated by 50% the positive association observed here between ART use and HTN. To the best of our knowledge, this is the first study to report the mediation effect of BMI on the association between ART use with hypertension or diabetes as outcomes. Some studies have, however, studied the mediation effect of BMI on the associations between ART use and BP. Nduka et al. observed a mediation effect of adiposity (waist circumference, but not BMI) in the association between HAART exposure and SBP and DBP [27]. It is therefore suggestive that ART use may partially contribute to cardiometabolic disease through adiposity. Before the advent and widespread use of ART, HIV-associated wasting and a high catabolic state with weight loss were very characteristic of the clinical picture of HIV. This reversed as ART uptake expanded and weight gain became common in PLWH. The mechanisms through which HIV causes weight gain and consequently cardiometabolic disease are unclear; however, the effects of ART use have been implicated [48]. Earlier PIs [49] and later INSTI [28, 50, 51] ART have been shown to increase weight gain in PLWH.

The main strength of this study is its large sample size. While there have been studies on hypertension and diabetes in PLWH in Africa, relatively few studies have had a sample size as large as ours. However, it is not without some limitations. First, the cross-sectional nature of the study limits any causal inferences. Second, the absence of an HIV negative control group limits comparability and generalizability of these results. Third, the study did not account for some unmeasured potential confounders such as dyslipidaemia, physical inactivity, family history of HTN and T2DM and diet. Additionally, the presence of potential unmeasured confounding and measurement error in the regression modelling for assessing mediation could bias indirect and direct effect estimates. We minimized this by choosing a suitable mediation analysis framework with sensitivity analysis to measure the magnitude of unmeasured confounding on the observed results and how much of it will mitigate the mediation effects. Using fasting blood sugar as the diagnostic test did not permit us to distinguish between participants who had type I or type II diabetes mellitus. However, only 1.4% of all participants with diabetes were less than 30 years old, the age group more prone to type I diabetes mellitus.

## Conclusion

These findings indicate that traditional cardiovascular risk factors, including older age, male sex, overweight and obesity, are strongly associated with HTN among PLWH. We also observed that BMI had a partial mediation effect in the association of ART use and HTN, but not T2DM. This study underscores the importance of screening, monitoring and management of HTN and T2DM particularly among older, male, and overweight/obese PLWH. Further research examining associations of HIV disease stage and ART use with HTN and T2DM are warranted.

## Data Availability

The data that support the findings of this study are available from the corresponding author upon reasonable request.

## Abbreviations

PLWH: People living with HIV
ART: Antiretroviral therapy
HTN: Hypertension
T2DM: Type-2 diabetes mellitus
BMI: Body mass index
SSA: Sub-Saharan Africa
IeDEA: International epidemiology Databases to Evaluate AIDS
CNERSH: Comité National Pour La Recherche en Sante Humaine
SBP: Systolic blood pressure
DBP: Diastolic blood pressure
ESC: European Society of Cardiology
ESH: European Society of Hypertension
FBG: Fasting blood glucose
WHO: World Health Organisation
LRT: Likelihood ratio test
ACME: Average causal mediation effect
ADE: Average direct effect
AGReMA-SF: Guideline for Reporting Mediation Analyses Short-Form
NRTI: Nucleoside reverse transcriptase inhibitors
NNRTI: Non-nucleoside reverse transcriptase inhibitors
INSTI: Integrase strand transfer inhibitors
PI: Protease inhibitors
OR: Odds ratios
HAART: Highly Active Antiretroviral Therapy
